# Supplementary material for: Causal Relationship between Aging and Anorexia Nervosa: A White-Matter-Microstructure-Mediated Mendelian Randomization Analysis
Source: Biomedicines. 2024 Aug 16;12(8):1874. doi: 10.3390/biomedicines12081874 (PMC11351342; doi:10.3390/biomedicines12081874)
Supplement: Supplementary file 1 [file biomedicines-12-01874-s001.zip › Supplementary Figure/Figure S4/Singe SNP plot of MR analysis for TL to ubm-b-2043.pdf]

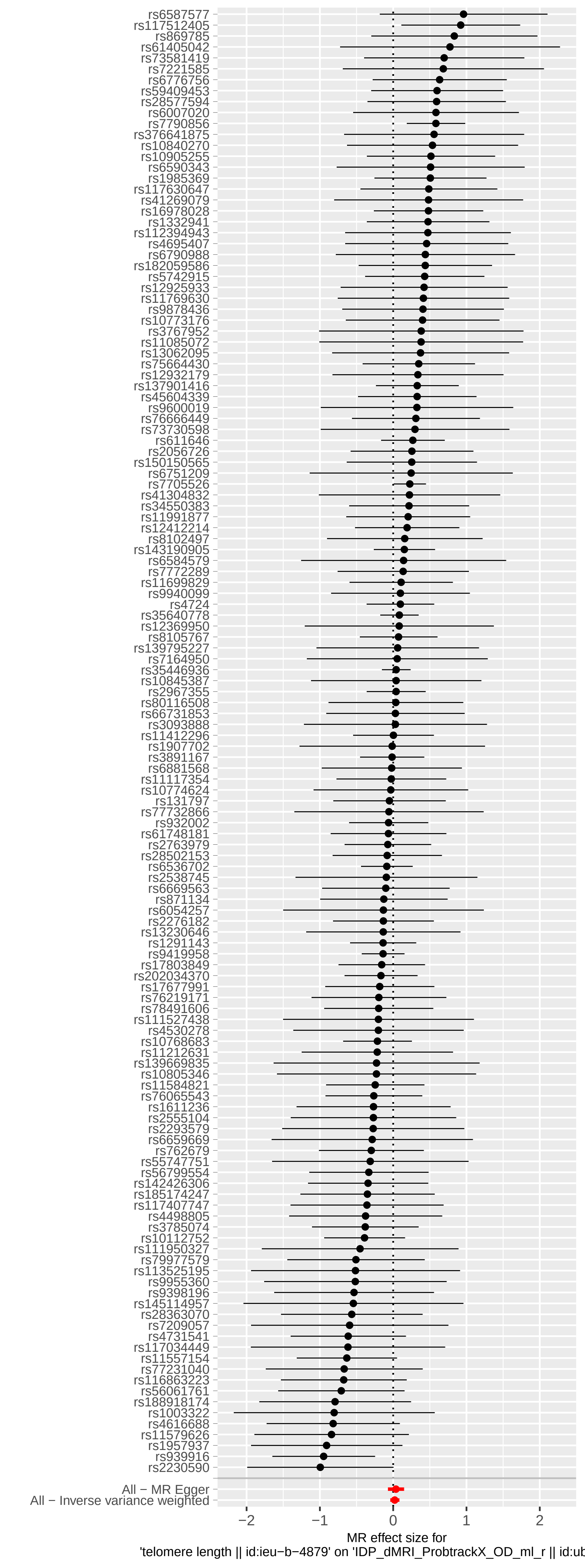

rs6587577
rs117512405
rs869785
rs61405042
rs73581419
rs7221585
rs6776756
rs59409453
rs28577594
rs6007020
rs7790856
rs376641875
rs10840270
rs10905255
rs6590343
rs1985369
rs117630647
rs41269079
rs16978028
rs1332941
rs112394943
rs4695407
rs6790988
rs182059586
rs5742915
rs12925933
rs11769630
rs9878436
rs10773176
rs3767952
rs11085072
rs13062095
rs75664430
rs12932179
rs137901416
rs45604339
rs9600019
rs76666449
rs73730598
rs611646
rs2056726
rs150150565
rs6751209
rs7705526
rs41304832
rs34550383
rs11991877
rs12412214
rs8102497
rs143190905
rs6584579
rs7772289
rs11699829
rs9940099
rs4724
rs35640778
rs12369950
rs8105767
rs139795227
rs7164950
rs35446936
rs10845387
rs2967355
rs80116508
rs66731853
rs3093888
rs11412296
rs1907702
rs3891167
rs6881568
rs11117354
rs10774624
rs131797
rs77732866
rs932002
rs61748181
rs2763979
rs28502153
rs6536702
rs2538745
rs6669563
rs871134
rs6054257
rs2276182
rs13230646
rs1291143
rs9419958
rs17803849
rs202034370
rs17677991
rs76219171
rs78491606
rs111527438
rs4530278
rs10768683
rs11212631
rs139669835
rs10805346
rs11584821
rs76065543
rs1611236
rs2555104
rs2293579
rs6659669
rs762679
rs55747751
rs56799554
rs142426306
rs185174247
rs117407747
rs4498805
rs3785074
rs10112752
rs111950327
rs79977579
rs113525195
rs9955360
rs9398196
rs145114957
rs28363070
rs7209057
rs4731541
rs117034449
rs11557154
rs77231040
rs116863223
rs56061761
rs188918174
rs1003322
rs4616688
rs11579626
rs1957937
rs939916
rs2230590
All − MR Egger
All − Inverse variance weighted
−2
−1
0
1
2
MR effect size for
'telomere length || id:ieu−b−4879' on 'IDP_dMRI_ProbtrackX_OD_ml_r || id:ub
